# Why we still perform arthroscopy in knee osteoarthritis: a multi-methods study

**DOI:** 10.1186/s12891-015-0537-y

**Published:** 2015-04-12

**Authors:** Timothy Barlow, Caroline Elizabeth Plant

**Affiliations:** Clinical Research Fellow, Warwick Medical School, Clinical Sciences Research Laboratories, Warwick Medical School, University Hospital of Coventry and Warwickshire, Coventry, CV2 2DX UK

**Keywords:** Knee osteoarthritis, Arthroscopy, Theoretical domains framework

## Abstract

**Background:**

Knee arthroscopy has historically been a common treatment for knee osteoarthritis. However, multiple Randomised Controlled Trials along with a Cochrane review has led NICE to recommend that arthroscopy is not used in the vast majority of patients that have knee osteoarthritis. These recommendations have been replicated internationally.

The use of arthroscopy for knee osteoarthritis has decreased; however, it is still prevalent. This study examines the factors that are perceived to influence decision-making using a theoretical framework that was developed for behaviour change research (Theoretical Domains Framework). This study will allow future work to develop and evaluate an intervention specifically targeted to the barriers identified.

**Methods:**

A multimodal approach was used including questionnaire research and semi-structured interviews with all grades of physician offering a knee arthroscopy service in a Level One Trauma Centre in the West Midlands, U.K. Focus groups with patients were also conducted. Mixed methods analysis was used, with descriptive statistics for quantitative data, and thematic content analysis for qualitative data.

**Results:**

A total of 26 surgeons responded to questionnaires, with 6 semi-structured interviews taking place. All surgical grades were represented. Two focus groups of six patients were performed. The results identified 13 beliefs across 12 domains (some beliefs were represented across domains). The beliefs that there was a pressure from patients to do something, that there were limited other options available, that surgeons wanted to meet patients expectations, and that there was a time pressure in clinic appeared to be the predominant barriers.

**Conclusions:**

Using the Theoretical Domains Framework, this paper has described the relevant barriers and enablers to the implementation of NICE guidance regarding arthroscopy use in patients with knee osteoarthritis. The next step in this process is the development of a targeted intervention, and we discuss the barriers that are most likely to alter practice if targeted through an intervention, and how such an intervention could look. Such an intervention would require evaluation within the clinical setting.

**Electronic supplementary material:**

The online version of this article (doi:10.1186/s12891-015-0537-y) contains supplementary material, which is available to authorized users.

## Background

Knee osteoarthritis (OA) is a common condition, affecting more than 10% of the population over 60 years old [1]. Knee arthroscopy has traditionally been a common tool in the treatment of knee OA. However a well publicised study by Moseley et al. [2], combined with a Cochrane review of the literature up to 2006 [3], has resulted in NICE guidance recommending that arthroscopy should not be used in knee osteoarthritis [4]. This guidance, updated in 2014, was based on “Gold” level evidence demonstrating a high cost and no clinical benefit of arthroscopy compared to conservative or placebo treatment [5].

Subsequent to the publication of the Moseley et al. paper, and the production of guidance both in the U.K. and internationally [4,6], there has been a decrease in the volume of knee arthroscopies performed for knee osteoarthritis internationally [7]. However, the data from such papers still suggests a significant number of arthroscopies are being performed for osteoarthritis. A major factor is the widespread criticism of the Moseley et al. paper, which resulted in poor acceptance of the results [8-11]. However, the criticism of the Moseley paper cannot be the only factor in the poor implementation of guidelines because subsequent randomised controlled trials and systematic reviews have been published that demonstrate consistent results [12]. Therefore, it is likely to be a multifactorial issue operating at the level of surgeon-patient interaction.

In response to the internationally poor adoption of guidelines, we conducted a region wide multi-centre audit within the West Midlands, aimed at University Teaching Hospitals with Level One Trauma Centres status. This demonstrated over 100 arthroscopies in the study centre being performed each year that were not in line with current guidelines. (Results unpublished, available on request). The guidance has had over five years to be implemented into clinical care; therefore, there are barriers to implementation.

There are a number of systematic reviews and meta-analysis on the effect different implementation strategies have on behaviour change. While these studies point to the limited evidence base of targeted interventions compared to others (e.g. audit and feedback), there appears to be a greater effect when a theoretically based targeted intervention is used [13,14].

This article describes a multimodal approach to understanding surgeons’ behaviour when listing patients for knee arthroscopy. This approach was based in the Theoretical Domains Framework (TDF) [15], which is a theoretical framework developed to aid implementation strategies within healthcare. It has been validated and used in multiple healthcare settings to assist with the systematic identification of barriers and enablers to implementation [16]. This study aims to understand the individual and institutional barriers and enablers as the first step in developing a targeted intervention to change practice. The next step, namely the development and evaluation of the effectiveness of the intervention, is not dealt with in this report.

## Methods

This was a multimodal study using an internet-based questionnaire with surgeons listing patients for arthroscopy, 6 in-depth interviews with the same surgeons, and two focus groups with patients. Such a mixed-methods approach is embedded in the MRC Framework for evaluating complex interventions, and allows a pragmatic approach to addressing the objectives of an implementation study while involving all stakeholders [17]. The questionnaires allowed a quantitative analysis of viewpoints, and allowed the generation of the range of views prevalent in the study population. This informed the development of the in-depth interview schedule, based on the TDF, which allowed further exploration of topics to a depth difficult to achieve with a questionnaire. A description of each TDF domain can be found in the results section (Table 1).

**Table 1:** **Summary of beliefs broken down by TDF domain**

**Domain**	**Specific Belief**	**Sample quote**
**Knowledge**	25% not aware of Randomised Controlled Trials or NICE guidance	Based on Questionnaire data
**Skills**	Resisting pressure of patients who want an arthroscopy	*Sometimes useful as a delaying tactic when under pressure from patient with minimal change but very symptomatic. (Questionnaire Participant 1)*
	Diagnosis of OA knee (WB film rather than non-WB or MRI)	“it [non-weight bearing radiographs and MRI] give you some leeway to offer what you want” (Interview participant 3)
**Social/Professional Role and Identity**	Resisting pressure of patients who want an arthroscopy (Professional confidence)	“Pressure from patients who do not want major surgery but want “something” done.’ (Questionnaire participant 6)
		“Expectation of patients to have a treatment/procedure prior to receiving arthroplasty.”(Questionnaire participant 7)
	Junior under pressure from seniors	“He is the boss” (interview participant 1)
		“It’s…. Commonly instigated by a senior surgeon” Interview participant 3)
**Beliefs about Capabilities**	Resisting pressure of patients who want an arthroscopy	“Pressure from patients who do not want major surgery but want “something” done.’ (Questionnaire participant 6)
		“Expectation of patients to have a treatment/procedure prior to receiving arthroplasty.”(Questionnaire participant 7)
	Belief that some surgeons better than average at arthroscopy, and will therefore have better results	“some surgeons do feel that they are better than average” (Interview participant 1)
**Beliefs about Consequences**	10% of respondents disagreed with NICE Guidance (most common in patients with mechanical symptoms); widely held belief that arthroscopy delays the need for TKR, and improves outcome in patients with knee OA	*Delay in treatment:*
		*“Sometimes useful as a delaying tactic when under pressure from patient with minimal change but very symptomatic.” . (Questionnaire Participant 1)*
		*“It makes it look like the knee replacement was delayed” (Interview participant 1)*
		*Subgroups of patients that may benefit:*
		*“The guidelines restrict treatment for the sub-group of patients who DO benefit from arthroscopic treatment in OA, or are unfit or do not wish to have more major interventions.” (Questionnaire participant 5)*
**Reinforcement**	Financial and regulatory factors (restrictions from commissioning groups and private insurance companies)	*“I think BUPA are getting independent reviews on private patients.”(Questionnaire participant 2)*
		*“Diagnostic arthroscopy alone is not a sufficient indication for surgery in the trust and it is not funded by the PCT. Therefore the patient is removed from the waiting list.” (Questionnaire participant 3)*
**Intentions**	Disagreement with guidelines result in no intention to adhere to them Stable	*“Patients cannot … be rigidly boxed into a protocol and clinicians, especially at consultant level, should have the freedom to assess patients on an individual basis.” (Interview participant 5)*
**Goals**	Returns to beliefs about outcome	*“Treat patients, not NICE guidelines. NICE guidelines assume patients are similar to machines, with no emotional input.” (Questionnaire participant 4)*
**Environmental Context and Resources**	Financial and regulatory factors	*“If all my peers stop doing it I would think twice, thrice, before offering it so yes, it would make me less likely to offer it.” (Interview participant 4)*
	Enabler is if other surgeons in department are not doing it	
	Resource issue	*“We essentially cant offer anything in the intermediate stage”(Interview participant 2)*
	Time pressure	*“You need more time to explain to patients what the option are if you are not doing arthroscopy”(Interview participant 1)*
**Social Influences**	Enabler if other surgeons in department are not doing it	*“It is harder to do a treatment none of your peers are.” (Interview participant 2)*
	Financial and regulatory factors	*As above*
**Emotion**	Desire to help – wanting to list even though might not be best thing (wanting to do something)	*“You do not want to dismiss their concerns” (Interview participant 1)*
		*“You want to help” (Interview participant 2)*
**Behavioural Regulation**	Just habit and learned behaviour that is driving the high arthroscopy rate	*“its been an established kind of solution for a long time … that is still a bit of a problem” (Interview participant 2)*
		*“Different consultants have different ways of managing … it would be a treatment they still would offer” (interview participant 1)*

Focus groups with patients were conducted after both of these stages to investigate specific barriers that were identified during the first two stages of the study. The design of each aspect is dealt with in turn.

Questionnaire research took place in November and December 2012, with subsequent interviews taking place from August 2013 to February 2014. Focus groups took place in November 2013 and February 2014.

### Questionnaires

The online questionnaire was developed after consultation with key stakeholders including senior orthopaedic surgeons and can be found in the supplementary material (Additional file 1) [18]. Issues that were thought to be relevant were populated into either free text questions or likert scale questions as appropriate. Development of the questionnaires was informed by NICE policy documents on implementation of guidance [19]. This approach allowed the development of a brief and targeted questionnaire aimed at quantifying various domains, alongside free text boxes. The questionnaire was piloted on two surgeons working externally to the study institution. This process did not alter the topics being covered, but resulted in some minor alterations in wording.

Surgeons who listed patients for knee arthroscopy in a University Teaching Hospital (n = 36) were sent a questionnaire via an online link [18]. Surgeons that received the questionnaires included all orthopaedic Consultants, Registrars, Associate Specialists, and Staff grades who were able to list patients for knee arthroscopy. No other practitioners in the study site were able to list patients for arthroscopy.

Surgeons were invited to participate via email, with one reminder email sent. All responses were then analysed with the display of descriptive statistics (proportions and percentages) where appropriate. Inferential statistics were not used. Open text answers were analysed using a thematic analysis that fed into the subsequent development of the in-depth interview schedule. The open text responses were also included in the analysis of the interviews, mapping themes to the TDF. A second researcher cross referenced 10% of this analysis (CP).

### In-depth interviews

Participants were orthopaedic surgeons who listed patients for knee arthroscopy. The aim of the purposive sampling procedure was to ensure a complete range of grades of surgeon. Sampling continued until all grades had participated in at least one interview, and at least three consecutive interviews had been conducted that did not add new material (i.e. saturation was achieved). Key opinion leaders, such as the Clinical Director, were specifically targeted as it was thought that their managerial role may lead to differing viewpoints.

Participants were approached individually and asked to take part in an interview. All surgeons that were approached agreed to take part and were interviewed by the lead author (TB) at the surgeons’ place of work. TB has experience in qualitative research methodology, conducting in-depth interviews, the clinical background, and the theoretical aims of the project. Due to technical constraints three interviews were unable to be recorded. Concise notes were taken in these instances. Transcripts of recorded conversations were transcribed, with the date of the interviews added to allow data tracking. All interviews lasted between 25 and 40 minutes.

The interview guide, broken down by TDF domain, is attached for information (see Additional file 2); however, it was used as a guide allowing the interviewee to explore areas in the order they preferred and at their own pace. The interview guide was piloted on two surgeons that did not work in the institution studied.

### Focus groups

Two focus groups were conducted with patients who had received a knee replacement for knee osteoarthritis in the last 12 months at the study site. The aim of this sample was to generate variation in our sample between those patients who had not had arthroscopies and those who had. Any sensitive issues raised with this group of patients were likely to have fewer repercussions for the patients as they had already progressed through their treatment for knee osteoarthritis.

Patients were recruited by an opt-in procedure with a letter and a study information sheet sent by post. 80 patients were contacted. Eligible patients who contacted the study co-ordinator were provided with further information and invited to participate in a focus group. Written consent was obtained for all participants.

The focus groups were designed to answer specific questions identified through the first two stages of the study, therefore the full set of TDF domains were not explored. They were conducted by a trained qualitative researcher with previous experience of facilitating focus groups (TB), with a patient representative present (PS) and another experienced qualitative researcher (AA). The focus groups were tape recorded and transcribed for analysis. Transcripts were sent to participants for review.

### Analysis of qualitative data

A coding framework was devised from the initial questionnaire data and mapped to the TDF. This allowed the subsequent development of the interview schedule. This process was conducted by TB. The interview data, together with the open text questionnaire data and focus group data was subsequently coded using a coding framework that mapped important domains (those that were mentioned frequently or seemed important to the participant) to the TDF. [20] Quotations were used to illustrate salient points. This thematic analysis, aimed at identifying common ideas within the data, was used across all sources of information (i.e. open ended questions from questionnaire data, transcribed interviews, and interviews that did not have verbatim transcription). Cross-referencing of 10% of the data analysis was performed by an independent researcher (CG). No disagreements on important domains were found; however, there were instances of disagreement over which domain certain ideas mapped to. This was resolved by discussion, or including the belief in both TDF domains. Saturation was deemed to have been reached when no new beliefs emerged with three successive interviews.

TB is an orthopaedic surgeon in training, and was a colleague of the orthopaedic surgeons involved in the research. The implications of this are explored in the discussion section.

### Ethical and institutional approval

Institutional approval was gained for questionnaire and in-depth interview stages of this study (University of Warwick Biomedical and Scientific Research Ethics Committee (BSREC) and the University of Coventry and Warwickshire NHS Trust Research, Development and Innovation department). Further approval was gained by the Dyfed Powys Research Ethics Committee (13/WA/0140) for the focus groups.

## Results

At the time of the study there were 11 consultants listing patients for knee arthroscopy, 18 registrar level trainees, four clinical fellows, and three Staff grade surgeons. The result section is split into three sections, and linked to the most relevant domain of the TDF where appropriate:

1. The descriptive statistics from the questionnaire research.
2. The finding from the qualitative analysis of the questionnaires, in-depth interviews, and focus groups linked to the TDF.
3. A succinct summary of the main findings.

Where appropriate some TDF domains have been reported together. This allows a more succinct report of findings and demonstrates where two domains are thought to be acting together.

### Descriptive data on questionnaire data

Table 2 demonstrates the number of responses generated for each grade. The overall response rate was 78%.

**Table 2:** **Tesponse rates**

**Grade of surgeon**	**Number sent survey**	**Response rate (percentage)**
**Consultant**	11	9 (81%)
**Staff grade/associate specialist**	3	0 (0%)
**Registrar**	18	17 (94%)
**Clinical Fellows**	4	2 (50%)
**Total**	36	26 (78%)

A technical issue resulted in some consultant surgeons who did not do knee arthroscopy procedures receiving the questionnaire. These surgeons either did not complete the questionnaire, or, in two cases, completed it. These questionnaires have been deleted from all analysis.

#### Knowledge

25% of respondents were not aware of the evidence base, or the NICE guidelines for arthroscopy in knee osteoarthritis.

#### Beliefs about consequences/Intention/Goals

10% of respondents disagreed with the NICE guidance, with 29% neither agreeing nor disagreeing. Disagreement was most common with regards to patients with mechanical symptoms. A widely held belief (32%) was knee arthroscopy delays the need for knee replacement, and that it improves outcome in patients with knee OA (21%).

#### Skills/Beliefs about capabilities /Social or Professional Role and Identity

One third of respondents felt under pressure to offer a surgical procedure other than knee replacement to patients. It was unclear from the questionnaire alone if patients actually wanted an arthroscopy, and if this pressure was perceived or real.

#### Skills/Beliefs about consequences

Half of respondents felt that using arthroscopy to treat patients with OA knee and mechanical symptoms, but no locking, was appropriate – this could be considered as half of respondents disagreeing with NICE guidance (see acceptance and belief).

One third of respondents would be happy proceeding to arthroscopy on the basis of a non-weight bearing radiograph +/− an MRI *when OA is a possible diagnosis.* (MRI and non-weight bearing radiographs do not have the required sensitivity to diagnose OA) [21].

#### Environmental context and resources

Financial and regulatory factors (mainly restriction in place through commissioning groups and private insurance companies) were reported as a factor that limited the number of arthroscopies in patients with OA knee. It was unclear if this was more a concern in the private sector or within the NHS.

### Qualitative analysis on interview and questionnaire data

Overall 6 interviews were conducted with medical staff (three consultants, one Registrar, one associate specialist, and one clinical fellow). This represented all of the relevant grades and included consultants that had managerial and teaching roles. As such this population represented the range of specialists referring patients for arthroscopy.

The analysis identified 12 domains that were implicated in listing patients for arthroscopy. This reflected 13 individual beliefs identified (some beliefs carried across various domains). The beliefs that carried across domains were the ones that were mentioned most frequently, and were felt to have the biggest effect.

Table 1 demonstrates a summary of the specific beliefs that were expressed, broken down by TDF domain.

#### Domains that affected decision making

Within the questionnaire research it was demonstrated that 25% of respondents were not aware of the evidence base, or the NICE guidance (TDF *Knowledge*). However, within the interviews all participants were aware of the indications for arthroscopy, although some were unable to describe the evidence base. The implication for the validity of the questionnaire findings are discussed in a subsequent section. Two beliefs were identified under the *Skills* domain. Firstly, diagnosis of OA knee using sub-optimal investigations (i.e. non-weight bearing radiographs or MRI scans c.f. weight bearing radiographs) was identified as prevalent within the questionnaire (one third) and as a factor within the interviews. However, within the interviews there was a sense that this was neither a skill or knowledge deficit that was present, but rather patients who had MRI and non-weight bearing radiographs that did not demonstrate OA meant that organising an arthroscopy was not specifically contraindicated. In this situation “chasing” a diagnosis of OA with further investigations was seen as then limiting the choice of treatment because an arthroscopy would then be harder to justify.

Secondly, and more importantly, there was the ability to resist pressure from patients who want an arthroscopy. This perceived pressure also maps to *Social/Professional Role and Identify* and *Beliefs about Capabilities*. It was impossible from the interviews to establish if this pressure was perceived or real, but added to it was the additional belief mapped to the *Emotion* domain of a genuine desire to help. Here, all surgeons interviewed expressed a desire to help their patients, a desire to leave the patients happy (and therefore meet their expectations and, by implication, accede to their wishes). Asked specifically about conservative management meeting expectation, many surgeons expressed the view that patients had already been down that road and expected something different. Combined with this idea is the prevalent belief, seen both within the questionnaires and the interviews, that certain subgroups of patients may indeed benefit (TDF *Beliefs about Consequences* and *Intentions).* This benefit could be in terms of improved outcome, but was more commonly expressed as a delay for the need for a knee replacement. Interestingly this delay was felt by some surgeons to be purely a system phenomenon (the waiting list for arthroscopy, combined with the recovery period post operatively “delayed” patients for up to a year). Put simply there is a proportion of surgeons who believe that some patients may benefit from arthroscopy, and all surgeons strongly hold a desire to help, and to satisfy, their patients. With the universally held belief that surgeons were under pressure from patients, all these factors appeared to be strongly held reasons for referral for arthroscopy.

A belief that some surgeons think that they are better than other surgeons was mapped to the *Beliefs about Consequences* domain. This would mean that results from pragmatic Randomised Controlled Trials would not necessarily apply to them, as they are better than the average surgeon. Interestingly, no surgeons thought that this applied to their practice, but felt that some colleagues may have this view. We were unable to demonstrate that this view existed within any member of the study; however, due to the nature of the issue, it would be difficult to gain definitive evidence. What was of interest is that those surgeons who identified this as a factor did not feel it was a significant cause of referrals for arthroscopy.

Financial and regulatory factors were identified as a factor in decision-making within questionnaires, and this was reflected in the interviews (TDF *Reinforcement* and *Environmental Context and Resources*). However, the influence this played in decision making was not universally accepted, and those that did acknowledge it felt it was of greater importance in the private sector.

A separate belief within *Environmental context and resources* was that there were little other options available for surgeons to offer patients. This was not a knowledge gap as to the other options that were available, but rather a view that there was no specific service that these patients could be referred to that would provide non-surgical care.

Time pressure was a factor that was also universally expressed (TDF Environmental Context and Resources). Within clinic it was felt that offering an arthroscopy was a quick consultation, relieving time pressure in clinic. Another factor within this domain was the enabler of departmental practice (also linked to *Social Influences*). It was felt that if surgeons in the department are not referring patients with OA for arthroscopy, this would discourage the practice department-wide.

The final belief, mapped to *Behavioural Regulation*, was that some surgeons have habit and a learned behaviour that is driving the high arthroscopy rate. As arthroscopy has been used in these patients for a long time the behaviour has been entrenched, and is not amenable to a quick change. An interesting offshoot from this was a belief held within the more junior surgeons interviewed. A further pressure that was perceived by them was that of the consultant-trainee interaction. Junior surgeons felt that if a consultant listed a patient for an arthroscopy there was a limited amount of influence they could exert on the situation (TDF *Social/Professional Role and Identity*).

#### Domains that did not affect decision-making

The Domains Memory, Attention, and Decision Process, and Optimism did not appear to have a large affect on decision-making. Specifically, cognitive overload or tiredness (especially when linked with time pressure in clinic) was not thought to be a major factor in the decision making process.

### Focus groups – what do patients want, and do they exert pressure on surgeons?

The questionnaire and interview study was unable to identify if the pressure from patients was real, or if patients even wanted a surgical procedure, therefore this aspect was designed to address this question.

12 participants took part in the focus groups, with four males, eight females. Participants were predominantly White British, reflecting the catchment area demographic, although one British Asian took part. Age ranged from 55 to 89, reflecting the typical ages that patients have knee replacements. Eight participants had previously had an arthroscopy. The focus groups had defined questions surrounding the pressure that patients exert on surgeons, and what they want. These focus groups took place at the Level One Trauma Centre under study. Both focus groups took two hours; however, decision making surrounding knee operations was also explored as part of another study during this time.

We found that patients who presented to an orthopaedic surgeon wanted something, but did not have a clear idea of what that was. The overpowering and universally held belief was that they were in pain and they wanted help. The form that help took was unimportant, and the desire to have an arthroscopy was not present in our study population (unless the *surgeon* thought it would help). Patients desired a more paternalistic interaction, with the surgeon prescribing the most appropriate method of care.

“[and the surgeon said] “So what do you want us to do?” and I’m thinking, “Hang on, mate, surely that’s your decision because you’re the professional here, and I’m looking to you for advice, I’m looking for you to lead me on the next part of my journey.” (Participant 1)

The perceived ability of patients to pressure surgeons into a particular course of action contrasted quite dramatically with the surgeons view: the majority of patients felt strongly that they would not be able to influence a surgeon’s action, as they would decide what was best (this correlated closely with the desire for a more paternalistic interaction).

“How can I as a total layman expect, it would be a cheek to even attempt to change his opinion” (Participant 7)

Some patients believed they could exert some pressure, but would not be able to alter decision making to a large extent (and certainly not be able to “get” an arthroscopy if it was not indicated).

Patients were asked specifically if a conservative management plan, with the core NICE recommended treatments (exercise, weight loss, and analgesia) would meet their expectations of treatment. There was widespread acceptance of this idea.

### Summary of main findings

There appear to be several key factors acting in concert that affect decision making, combined with several factors that are likely to have a minimal effect.

Factors that have less of an effect include the belief that patients may benefit from an arthroscopy (either by a system process that delays knee replacement, or by certain sub-groups benefiting) may be present. Although reasonably prevalent it would not apply to a large proportion of patients (only certain sub-populations). It would also not be particularly amenable to intervention as the viewpoint was present in surgeons who appeared to have a good grasp of the current literature. Beliefs over pressure on juniors from consultants, and ordering the appropriate investigations for OA appear to be quite complex. It is likely that these factors would be addressed if the underlying behaviour of referral to arthroscopy was altered (e.g. appropriate investigation such that if a diagnosis of OA is made it does not seem to be limiting treatment options). This argument would also apply to both learned behaviour as a driving force for referrals. Regulatory factors and social influences in the form of other surgeons not performing such arthroscopies acting as a disincentive could be viewed as enablers. This would suggest that any intervention that successfully altered some consultants’ referral behaviour would have an effect on others.

The major barriers that drove referrals for arthroscopy acted in concert. A desire to help patients and meet their expectations, a belief that those expectations did not involve conservative measures (and there was no service available that offered such conservative measures), time pressure in clinic, and a perceived (or real) pressure from patients for an arthroscopy all contributed to a substantial barrier. It is clear from the focus groups that this pressure is not specifically for an arthroscopy; however, patients do want “something” done.

## Discussion

We have identified the most relevant factors affecting decision-making when surgeons refer patients for arthroscopy in knee OA. The use of the TDF and the rigorous application of multiple methods have enabled us to identify which of these factors would be the most appropriate, and likely to lead to the biggest change, if targeted in an intervention. To our knowledge, this is the only study of its kind examining decision making in this setting.

This study has used multiple methods, as recommended by the MRC, and has been based in behaviour change theory for use in healthcare models. This approach has allowed the systematic exploration of barriers and enablers, and allowed us to use each study methodology to answer specific questions. The iterative and reactive nature of the study allowed us to explore all aspects of decision making from both service users and service provider points of view. However, there are limitations in our study design. Not all people who were sent the questionnaire responded -- recent reports demonstrating the difficulties with online, opt in, surveys have been published [22], with some studies demonstrating a 22% response rate with this approach [23]. The non-responders may be systematically different to the responders – for example no staff grade or associate specialists replied to the survey. However, this population represents a small number, and with a total response rate of over 75%, we are confident that we obtained a relatively representative cohort. The results represent a snapshot of the current barriers to implementation, which does not allow for changes over time. Additionally the qualitative nature of the study will only determine the views of the participants. Therefore extrapolation to other centres, and through altered practice in the same centre, should be conducted with caution, or not attempted at all. The ability of the study to identify institutional issues, primary care issues, or integration of care issues, is limited by using the TDF and interviewing surgeons. This limitation is balanced against a good understanding of the barriers acting at a surgeon level, and although data was gathered at an individual level, some data on barriers acting at an institutional level can be gleaned from the TDF (e.g. the domain Environmental consequences and resources). All patients within our study had already had a knee replacement and therefore may not be best placed to discuss the treatment of early OA; however this allowed a discussion between service users who had not had an arthroscopy and those that had. The relationship between the interviewer and participants may have led to biases (interviewer and participants were colleagues). However, the interviewer’s relationship was also likely to lead to a good rapport and trust allowing interviewees to be more forthright in their opinions. This could explain the early achievement of saturation. Additionally, the questionnaire development process was limited, although multiple stakeholders were identified and the questionnaire was piloted. We judged that the use of such a questionnaire was justified as its purpose was to provide a snapshot of current opinion, and to help focus the subsequent stages of the investigation. We believe early saturation was achieved due to the focussed nature of our aim and the multi-modal approach used, and we are confident that any barriers were identified. The use of case notes as opposed to verbatim transcription for some interviews may lead to less reliable results; however, the remarkable consistency across all modes of study suggest this in not the case. A further limitation of this study is that it will not, by itself, lead to improved health care practices. Further action, aimed at addressing these barriers, is required to make a difference to the delivery of health care.

In 2012 French et al. described behaviour change strategies mapped to domains in the TDF. [22] This process has been tested previously, and targeted interventions do appear to have a larger effect on changing practice than generic, non-tailored interventions [23]. Using this framework will allow the development of a targeted intervention aimed at the barriers identified in this study. One of the benefits of this study has been the ability, through the use of multiple methods, to fully explore each of the domains of the TDF. This has enabled us to identify which domains would be best to target. Specifically, an intervention that: relieved time pressure in clinics (i.e. was as quick as listing for an arthroscopy); allowed the surgeons to feel that they were helping, meeting the patients expectations, and leaving the patient satisfied; and met with the patients aim of being offered something. An intervention that achieved this would, in theory, not only address these factors but also facilitate change by relieving pressure on juniors and facilitating change by social and environmental influences (if other surgeons are not doing it, the pressure to also not do it is increased). However, the belief that certain sub-groups of patients do benefit is an area where any intervention is unlikely to alter. This is due to the correlation of this belief overlapping with a good understanding of the NICE guidance and the various Randomised Controlled Trials conducted in the area. It is unlikely any intervention will improve the knowledge base, or alter behaviour for this sub-group of surgeons. However, as this only applies to the minority of patients, it is unlikely to have a dominant effect.

Similar studies, primarily in primary care, use interventions that would be consistent with the approach described above. For example an Australian study has developed a care pathway targeted at improving the use of conservative management of knee OA [24]. In the Netherlands the BART study developed a strategy for offering conservative management to patients [25]. Within the U.K., the MOSAIC study is looking to improve management of OA in primary care [26]. These studies suggest that more advanced options should be tailored to patients as they progress through their treatment. All patients in our study will have been through their General Practitioner and therefore a more advanced programme of conservative management would need to be considered. With specific relevance to the U.K. this intervention would need to complement any care given in Primary Care: depending on the results of the near completed MOSAIC study, integration of care to provide consistent and cost effective care pathway would be the ideal. A key aspect of such an intervention would be developing it with the predominant barriers in mind and targeting those barriers using a strategy similar to that proposed by French [22].

Within orthopaedics the BART-OP study is on-going in the Netherlands [27]. This is aimed at identifying the barriers to implementation of conservative management in OA, and includes secondary care. This study has a slightly broader context, as the aim is not to reduce arthroscopy rates, and is taking place in a different population and health care systems; however, we would expect the results from that study as regards the barriers to implementation to be similar.

## Conclusion

Using a multi-modal approach embedded in the TDF we have identified the most appropriate barriers to target in order to decrease the number of arthroscopies that are performed for patients with OA of the knee. These represent theoretically derived targets for future intervention strategies. These strategies are likely to complement strategies already developed, and currently being evaluated, in primary care.

## Additional files

Additional file 1:
**Arthroscopy in knee osteoarthritis.**
Additional file 2:
**Questions grouped by TDF domain.**
