# Supplementary material for: Why we still perform arthroscopy in knee osteoarthritis: a multi-methods study
Source: BMC Musculoskelet Disord. 2015 Apr 12;16:85. doi: 10.1186/s12891-015-0537-y (PMC4435528; doi:10.1186/s12891-015-0537-y)
Supplement: Additional file 1: — Arthroscopy in knee osteoarthritis. [file 12891_2015_537_MOESM1_ESM.pdf]

Arthroscopy in knee osteoarthritis

1. What grade of orthopaedic surgeon are you?

- [ ] Consultant
- [ ] Speciality Registrar
- [ ] Associate Specialist/Staff Grade

Other (please specify)

2. Are you aware of any RCT or systematic reviews that deal with knee arthroscopies in OA?

- ( ) Yes
- ( ) No

3. Are you aware of NICE guidelines on knee arthroscopies in OA?

- ( ) Yes
- ( ) No

4. Are the guidelines applicable to your practice?

Strongly disagree	Disagree	Niether agree nor disagree	Agree	Strongly agree
( )	( )	( )	( )	( )

5. If you are aware of the guidelines, do you agree with them?

Strongly disagree	Disagree	Niether disagree nor agree	Agree	Stongly agree
( )	( )	( )	( )	( )

6. Please explain your answer to question 5.

7. Do you think that knee arthroscopy delays the need for a TKR in knee OA?

Strongly disagree	Disagree	Niether disagree nor agree	Agree	Strongly Agree
( )	( )	( )	( )	( )

8. Do you think that knee arthroscopy improves outcome in patients with knee OA?

Strongly disagree	Disagree	Niether disagree nor agree	Agree	Strongly agree
( )	( )	( )	( )	( )

9. Do you feel under pressure to offer an operation to patients with knee OA who you do not think require a TKR?

Strongly disagree	Disagree	Niether disagree nor agree	Agree	Strongly agree
( )	( )	( )	( )	( )

Arthroscopy in knee osteoarthritis

10. Do you commonly use arthroscopy to diagnose OA knee?

Strongly disagree	Disagree	Niether agree nor disagree	Agree	Strongly agree
○	○	○	○	○

11. In patients with OA knee and no locking or mechanical symptoms, do you use arthroscopy to rule out other intra-articular pathology?

Strongly disagree	Disagree	Niether disagree nor agree	Agree	Strongly agree
○	○	○	○	○

12. Do you use arthroscopy to diagnose/treat patients with OA knee and mechanical symptoms, but no locking?

Strongly disagree	Disagree	Niether agree nor disagree	Agree	Strongly agree
○	○	○	○	○

13. Do you think arthroscopy has a place in 10 and 11 Above?

○ Yes

○ No

14. If yes, please expand on your answer.

15. In a patient where OA knee is a possible diagnosis, would a non-WB plain film +/- MRI sufficiently rule out significant OA, such that an arthroscopy could be appropriate?

Strongly disagree	Disagree	Niether disagree nor agree	Agree	Strongly agree
○	○	○	○	○

16. Are you aware of any financial incentive not to perform knee arthroscopy in patients with OA?

○ Yes

○ No

17. If yes, please expand on your answer.

18. Are you aware of any regulatory process in place designed to monitor knee arthroscopy in patients with OA?

☐ Yes

☐ No

19. If yes, please expand on your answer.

Arthroscopy in knee osteoarthritis

20. Do you feel there are practical issues that result in offering, and performing, arthroscopies in patients with knee OA?

☐ Yes

☐ No

21. If yes, please expand on your answer.
