# Supplementary material for: Why we still perform arthroscopy in knee osteoarthritis: a multi-methods study
Source: BMC Musculoskelet Disord. 2015 Apr 12;16:85. doi: 10.1186/s12891-015-0537-y (PMC4435528; doi:10.1186/s12891-015-0537-y)
Supplement: Additional file 2: — Questions grouped by TDF domain. [file 12891_2015_537_MOESM2_ESM.docx]

# Questions grouped by TDF domain

| TDF Domains | TDF Definitions | Questions |
| --- | --- | --- |
| Knowledge | An awareness of the existence of something. | Are you aware of the NICE guidance?  Are you aware of any Randomised controlled evidence regarding the use of arthroscopy in patients with Knee OA?  Are there subgroups that you would consider/not consider arthroscopy in? |
| Skills | An ability or proficiency acquired through practice. | What skills are needed?  Do you feel confident in identifying which patients require knee arthroscopy in OA?  How would you diagnose OA (Specifically MRI, non-WB film)?  Would you use arthroscopy in a patient who did not have mechanical locking and who had a diagnosis of OA? |
| Social professional role and identity | A coherent set of behaviors and displayed personal qualities of an individual in a social or work setting. | Do you feel it is your role to protect patients from procedures that do not help them?  If you agree with the NICE guidance, and with the statement above, how to you justify to yourself listing patients for knee arthroscopy?  Do you feel that it is ethical to use a placebo response on patients? |
| Beliefs about capabilities | Acceptance of the truth, reality, or validity about an ability, talent, or facility that a person can put to constructive use. | Are there any difficulties in not offering arthroscopy to patients?  Do you feel under any pressure from anyone (e.g. patients, colleagues, management) to offer or not offer arthroscopy? If so, how do you deal with that pressure?  Do you think that a surgeon who believes he is better than average, and will therefore have better results, and consequently list for arthroscopy? Do you identify with this in you or any colleagues? |
| Optimism | The confidence that things will happen for the best or that desired goals will be attained | How confident are you that offering an arthroscopy will result in the patient being satisfied with the consultation?  Do you think they are satisfied after the arthroscopy? |
| Beliefs about consequences | Acceptance of the truth, reality, or validity about outcomes of a behavior in a given situation. | What are the consequences of offering an arthroscopy?  And of not offering one? |
| Reinforcement | Increasing the probability of a response by arranging a dependent relationship, or contingency, between the response and a given stimulus. | Are patients who have an arthroscopy happy?  Does offering an arthroscopy result in an easier consultation? |
| Intentions | A conscious decision to perform a behavior or a resolve to act in a certain way | Do you list patients for arthroscopy knowing it is not in line with NICE guidance? |
| Goals | Mental representations of outcomes or end states that an individual wants to achieve | What do you want when a patient with knee OA presents to you? |
| Memory, attention and decision processes | The ability to retain information, focus selectively on aspects of the environment and choose between two or more alternatives. | Does feeling tiered or stressed mean that you are less able to deal with pressures from …. (link to beliefs about capabilities)? |
| Environmental context and resources | Any circumstance of a person’s situation or environment that discourages or encourages the development of skills and abilities, independence, social competence, and adaptive behavior. | Are there any other options for these patients? For example conservative management in line with NICE guidance?  Do you have enough time in clinic to address these issues?  If other surgeons are not preforming a surgical procedure in your department, does that make you more or less likely to perform the same procedure? |
| Social influences | Those interpersonal processes that can cause individuals to change their thoughts, feelings, or behaviors. | If other surgeons are not preforming a surgical procedure in your department, does that make you more or less likely to perform the same procedure?  Are you aware of any financial or regulatory barriers to performing arthroscopy in patients with knee OA? |
| Emotion | A complex reaction pattern, involving experiential, behavioral, and psychological elements, by which an individual attempts to deal with a personally significant matter or event. | Is not meeting patient’s expectations difficult?  Do you feel that by not offering an arthroscopy you are not meeting their expectations?  Would you prefer to offer patients something? |
| Behavioral regulation | Anything aimed at managing or changing objectively observed or measured actions. | Do you think that patients are referred out of habit?  Do you think there are any process in place to monitor or change your listing behavior? |
